# Evaluating the use of red flags by online symptom checkers

**DOI:** 10.1186/s12913-025-13353-w

**Published:** 2025-10-01

**Authors:** Shailen Sutaria, Delanjathan Devakumar, Poppy Mallinson, Sanjay Kinra, Tamer T. Malak, Andras Meczner

**Affiliations:** 1 Faculty of Epidemiology and Population Health, London School of Hygiene and Tropical Medicine, Keppel St, London, WC1E 7HT UK; 2 Institute for Global Health, 3rd floor, Institute of Child Health, University College London, 30 Guilford Street, London, WC1N 1EH UK; 3 Your.MD Ltd/Healthily, 167-169 Great Portland Street, 5th Floor,, London, W1W 5PF UK; 4 Flo Health Ltd Institute for Clinical Data Management, 27 Old Gloucester Street, London, WC1N 3AX UK; 5 Semmelweis University, Baross u 52, Budapest, 1082 Hungary

**Keywords:** Online symptom checkers, Evaluation, Red flags, Triage

## Abstract

**Background:**

Online Symptom Checkers (OSCs) are digital health tools providing triage, diagnostic, and self-care advice based on user reported symptoms. Amidst global trends of increasing demand and workforce shortages, OSCs have the potential to alleviate primary care workload. However, their ability to seek red flag symptoms, a critical marker of a safe consultation in primary care, remains unexplored. Using clinical vignettes, this study evaluates OSCs’ performance in seeking red flag symptoms compared to Primary Care Physicians (PCPs).

**Methods:**

Four OSCs (Ada, Babylon, Symptomate, Healthily) were evaluated using 51 clinical vignettes. Two standard setters used guidelines to determine which vignettes required emergency triage and identified the relevant red flags symptoms for the remaining vignettes. Two laypersons entered data from vignettes into OSCs and outputs were collected following a standardised form. The same vignettes were independently assessed by PCPs to compare triage accuracy and red flag identification. Summary statistics and 95% confidence intervals were calculated using Wilson Score intervals, and Fisher’s exact test was used to compare performance between OSCs and PCPs.

**Results:**

Of the 51 clinical vignettes, standard setters determined 14 to require emergency triage and the remaining 37 vignettes suitable for primary care triage. Of the primary care triaged vignettes, standard setters identified a total of 77 relevant red flag symptoms to be sought. Of the 14 emergency vignettes, PCPs correctly triaged 85.7% (95% CI: 74.3–92.6%) of cases compared to OSCs 76.9% (95% CI: 59.3–87.9%), with no statistically significant difference (*p* = 0.299). Specificity, the proportion of correctly triaged primary care vignettes, PCPs performed significantly better compared to OSCs, 91.9% (95%CI 78.9–97.0%) vs. 83.3% (95%CI 68.1–91.9%), *p* = 0.024.

**Conclusions:**

OSCs demonstrated comparable ability to appropriately triage clinical vignettes requiring emergency triage as PCPs, however, were less specific, triaging more primary care vignettes as emergency. OSCs do not seek the majority of red flags. This raises concerns about their safety and effectiveness in primary care. OSCs developers should focus on improving OSCs' red flag coverage to ensure safe integration into primary care settings.

**Supplementary Information:**

The online version contains supplementary material available at 10.1186/s12913-025-13353-w.

## Introduction

Online Symptom Checkers (OSCs) are digital health tools accessible via web and mobile applications that provide triage, diagnostic and self-care advice based on a user’s reported symptoms. Designed for public use, OSCs enable users to quickly obtain personalised health information without the need to engage with formal healthcare services. OSCs can aid in the triaging of patients to the appropriate healthcare setting, differentiating primary care and emergency care presentations and identifying users who can safely manage their symptoms at home through self-care advice [1]. Given the challenges of increasing primary care demand and workforce shortages facing primary care both worldwide and in the UK [2], OSCs may serve as a valuable adjunct in managing primary care workloads.

Traditional evaluations of OSCs have largely focused on diagnostic and triage accuracy as indicators of safety, with reported accuracies of 34–65% [3–5]. In primary care, a substantial number of consultations do not lead to a diagnosis, and one in five cases involve minor, self-limiting symptoms that require no further investigation [6, 7]. In this context, ruling out serious conditions is crucial to ensuring that rare but severe illnesses are not overlooked. One method that has emerged in clinical practice to do this is the use of ‘red flags’ [8]. Red flags are symptoms that are specifically elicited by primary care physicians (PCPs) to consider more serious illness. For example, in acute back pain, a common and self-limiting condition that accounts for 8% of primary care consultations [9], red flags are used to identify Cauda Equina Syndrome. This is a rare but serious cause of acute back pain that requires urgent investigation and management [10–12]. Therefore, diagnostic accuracy alone may not be an adequate measure of safety when assessing the use of OSCs in primary care. This nuance may be absent in OSCs and undermine their safety in a primary care context.

Two essential requirements of primary care involve the rapid identification of symptoms requiring hospital emergency care (triaging) and differentiating symptoms caused by benign self-limiting illness from those caused by serious illness [13]. 

To date, no studies have examined whether the current calibre of OSCs incorporate red flags symptoms. Evaluating their ability to seek red flags may provide a critical safety marker that goes beyond diagnostic accuracy and is relevant to primary care, where PCPs need to balance risk of investigations for mild illness against the risk of missing serious illness.

We aim to evaluate the ability of a selection of commercially available OSCs to (1) accurately identify individuals requiring emergency care and (2) evaluating red flags coverage compared to PCPs, in primary care setting.

## Methods

### Study design

We evaluated four popular, commercially available Online Symptom Checkers (OSCs) in the UK —Ada, Babylon, Symptomate, and Healthily based on their high performance in previous evaluations [14]. 

Using clinical vignettes to simulate patient consultations, we aimed to assess their ability to meet two critical needs in primary care: (1) triaging patients requiring same-day emergency care and (2) identifying relevant red flag symptoms among cases suitable for primary care.

### Simulated consultations

We aimed to recreate a typical on-call day for a PCP working in UK primary care. Study author (SS), a qualified and practicing primary care physician, selected 51 clinical vignettes to reflect the typical caseload of a PCP (Appendix 1, Table 1). The majority (90%) were obtained from previously published OSC evaluations [3, 15, 16]. The remaining 10% were obtained from a popular provider of clinical vignettes, used by UK doctors in the preparation of The Royal College of General Practitioners licensing exams [17].

**Table 1:** Number and percentage of correctly triaged emergency vignettes by OSCs and PCPs

Group		Emergency cases correctly triaged^c^		Non-Emergency (Primary care) cases correctly triaged^c^		Sensitivity (%)			Specificity (%)		
		Inputter 1	Inputter 2	Inputter 1	Inputter 2	Overall	Mean 95% CI^a^	OSCs vs. PCPs *P*-value^b^	Overall	Mean 95% CI^a^	OSCs vs. PCPs *P*-value^b^
OSCs	Ada	13/14	13/14	33/37	34/37	92.9%	76.9% (59.3–87.9%)	0.299	90.5%	83.3% (68.1–91.9%)	0.024
	Babylon	6/13	7/12	27/32	28/36	52.0%			80.9%		
	Healthily	9/12	12/14	19/29	26/33	80.8%			72.6%		
	Symptomate	11/14	12/14	34/37	32/37	82.1%			89.2%		
PCPs	PCP 1	12/14		33/37		85.7%	85.7% (74.3–92.6%)		89.2%	91.9 (78.9–97.0%)	
	PCP 2	12/14		33/37		85.7%			89.2%		
	PCP 3	11/14		36/37		78.6%			97.3%		
	PCP 4	13/14		34/37		92.9%			91.9%		

a = 95%CI calculated using Wilson Score interval

b = Fisher’s Exact Test

c= Denominator includes only vignettes where an outcome is given

Each vignette was entered into each OSC twice by two independent non-medical inputters, yielding 102 entries per OSC and 408 total outcomes. This dual-input approach was employed to reduce variability in data entry [18].

### Standard setting

Two medically qualified PCPs (MR, BM) were involved in standard setting (triage and red flag) and not involved in any other aspect of study design or testing. Both have over 15 years’ experience working in primary care settings in the UK, and BM is involved in medical education, including assessment and standard setting for medical students and health care professionals.

### Emergency and primary care triage

Standard setters (MR, BM) were asked to independently triage vignettes into “emergency care”, if requiring same day emergency care in a hospital setting or “primary care”. Results were combined and where differences emerged, external guidelines were reviewed, and consensus obtained.

### Red flags

For vignettes triaged to primary care, standard setters (MR, BM) collaboratively identified relevant red flags using clinical experience and relevant national guidelines. Red flags were defined for this study to be additional relevant symptoms that would be sought to exclude serious illness, requiring a change in management (such as urgent need for investigation). Red flags were not assessed for emergency care cases, as these would bypass primary care.

### Assessment of OSCs

Two laypersons (non-medical inputters), educated to secondary school level, were financially compensated for entering data into OSCs. Following a standardised inputter protocol, they independently entered all 51 vignettes into each OSC between August and December 2022. Input data, further symptoms asked by OSCs, and outcomes were recorded in OSC-specific spreadsheets. The researchers then compared symptoms asked by the OSCs with red flag symptoms for each specific vignette, to determine the number of relevant red flag symptoms sought per vignette by each OSC.

### Comparison

Independently, four PCPs, not involved in vignette selection or standard setting, currently working as PCPs assessed the same 51 clinical vignettes. Each PCPs was asked to assign the appropriate triage (emergency or primary care) and secondly among primary care triaged, the additional symptoms they would seek during a typical consultation without revealing the purpose of the study. The researcher then compared the symptoms asked with red flag symptoms for each specific vignette, determining the number of relevant red flag symptoms sought per vignette by each PCP.

### Analysis

We calculated summary statistics and 95% confidence intervals using Wilson Score interval for proportion of emergency triage correctly triaged (sensitivity), proportion of primary care cases correctly triaged (specificity), and proportion of red flags asked by OSCs and PCPs. We used Fisher exact test to compare overall OSC performance against overall PCPs performance in coverage of red flags and emergency triage accuracy.

## Results

### Standard setting

Of the 51 clinical vignettes, 14 were determined to be requiring same day triage to an emergency setting, with the remaining 37 suitable for primary care management (Appendix Table 1).

Of the 37 primary care triaged vignettes, 77 Red flags were identified, ranging from 0 to 8 red flags per vignette, mean 2.6 red flags per vignette. The majority (67.6%) of vignettes had 1 or more red flags (Appendix Table 2).

### Accuracy of triage of emergency care vignettes (Table 1)

Sensitivity was calculated as the proportion of the 14 emergency triage vignettes that were correctly identified. 14 Emergency triage vignettes were entered into each OSC by two different inputters, resulting in a total of 112 emergency vignettes of which 107 generated triage data. While all vignettes were entered, results were based on only vignettes where the OSC gave a triage recommendation. On average, OSCs correctly identified 76.9% (95%CI 59.3–87.9%) of the emergency vignettes, this ranged from 52 to 93% by individual OSC. On average, PCPs correctly triaged 85.7%, (95%CI 74.3–92.6%) of the emergency vignettes, ranging from 79 to 93% by individual PCPs. There was no statistical difference between PCPs and OSCs (*p* = 0.299).

Specificity, the proportion of non-emergency triage (primary care triage) correctly identified, 37 non-emergency care vignettes were similarly entered into each OSC by two different inputters, resulting in a total of 296 non-emergency triage vignettes. On average, OSCs correctly identified 83.3% (95%CI 68.1–91.9%) versus 91.9% (95%CI 78.9–97.0%) by PCPs, *p* = 0.024.

### Red Flags (Fig. 1; Table 2)

**Fig. 1:**
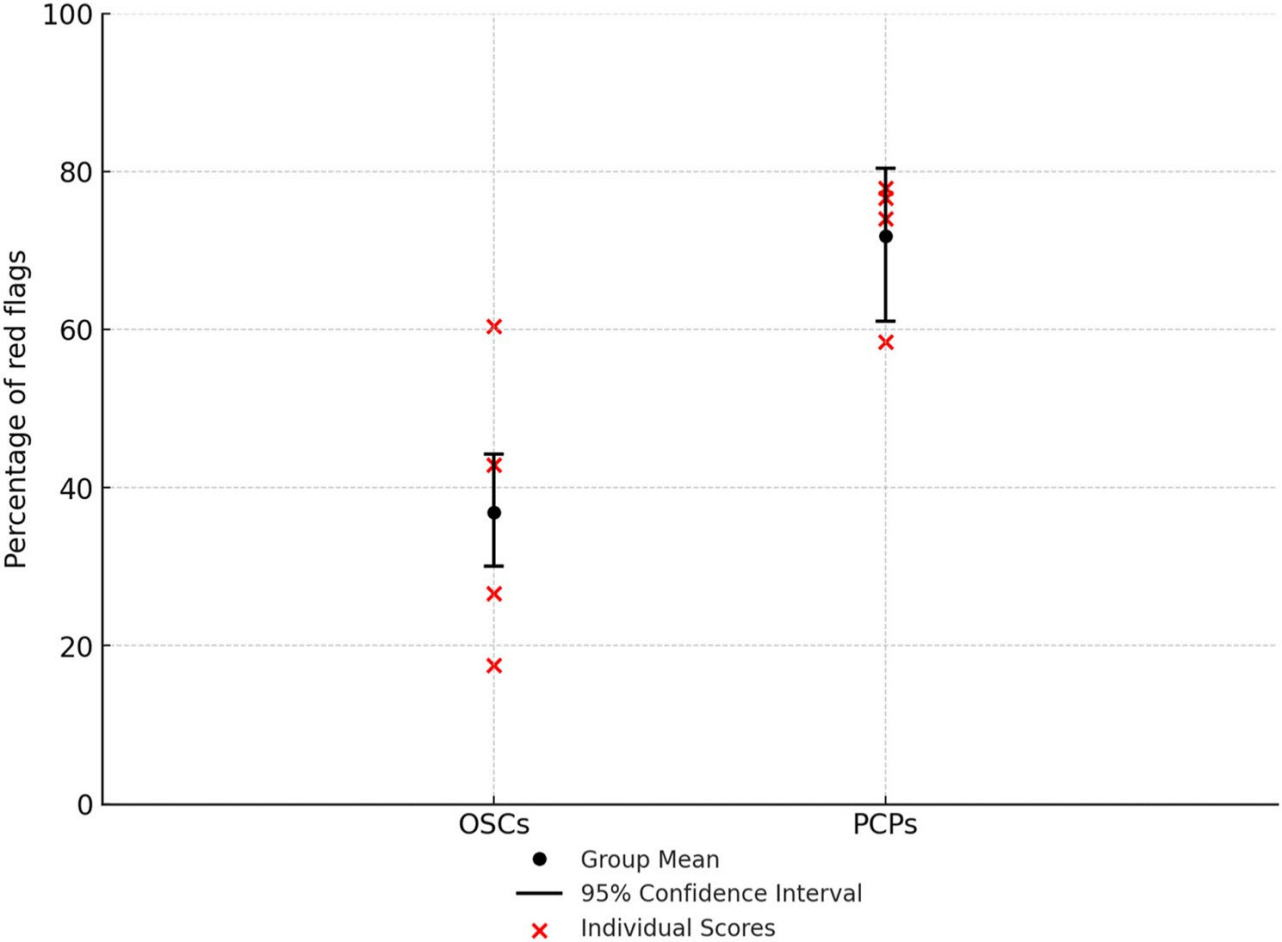
Percentage of total red flags identified by online symptom checkers and by primary care physicians

**Table 2:** Number and percentage coverage of red flags questions among primary care vignettes by inputter and OSC

Group		Inputter 1	Inputter 2	Overall (%)	Group Mean and 95% confidence interval (%)^a^	*P*-value^b^
OSCs	Ada	14/77	27/77	26.6%	36.9% (30.1-44.2%)	*p*<0.001
	Babylon	29/77	37/77	42.9%		
	Healthily	44/77	49/77	60.4%		
	Symptomate	9/77	18/77	17.5%		
PCPs	PCP 1	57/77		74.0%	71.8% (61.0-80.4%)	
	PCP 2	45/77		58.4%		
	PCP 3	60/77		77.9%		
	PCP 4	59/77		76.6%		

a = 95%CI calculated using Wilson Score interval

b = Fisher’s Exact Test

37 individual primary care vignettes with a potential of 77 relevant red flag to be sought were entered into four OSCs by 2 inputters resulting in 296 vignettes, and a total of 616 potential red flags to be sought. All 37 primary care vignettes were given to 4 PCPs, who were able to provide data for all vignettes given.

Overall, OSCs asked 36.9% of red flags symptoms (95%CI 30.1–44.2%), ranging from 17.5 to 60.4% by individual OSC. PCPs asked significantly more red flags compared to OSCs (*p* < 0.001), capturing 71.8% (95%CI 61.0-80.4%) of red flags ranging from 58.4 to 77.9% by individual PCPs.

## Discussion

Using clinical vignettes, we examined the ability of a sample of OSCs to perform two key aspects of primary care. Firstly, their ability to accurately triage emergency presentations and secondly their ability to ask relevant red flag symptoms compared to PCPs.

We found OSCs correctly triaged 76.9% (95%CI 59.3–87.9%) emergency vignettes compared to 85.7% (95%CI 74.3–92.6%) correctly triaged by PCPs, this difference was not statistically different (*p* = 0.299), however the specificity (ability to correctly triage non-emergency cases) was significantly lower that PCPs (83.3% vs. 91.9%, *p* = 0.024), suggesting that OSCs may be overly safe and triage more cases to emergency, this is similar to findings in previous studies [5]. OSCs asked significantly fewer red flags than PCPs (36.9% vs. 71.8%, *p* < 0.001), this may have been several reasons. PCPs can read clinical vignettes and capture all the information presented, enabling them to ask targeted additional diagnostic and red flag symptoms not already contained in the vignette. In contrast, inputters must choose and input specific symptoms from the vignettes into the OSCs, which then generate further questions. Thus, OSCs can miss vital information already contained in the vignettes if not initially entered or prompted by the OSCs. Feedback from inputters indicated variability in the number of questions asked by OSCs during consultations. Additional analysis on a subset of vignettes, revealed the average number of questions per vignette asked by OSCs, were 17.6, 21.5, 39.2 and 17.4 for Ada, Babylon, Healthily and Symptomate respectively. This may partially explain the difference in performance seen, with OSCs that ask more questions more likely to identify important red flag symptoms, however, this may come at the cost of user experience and time to completion. Developers of OSCs face the challenge of balancing thoroughness and user experience with excessively long consultations, risking user disengagement [19]. Advances in large language models may help address these limitations by enabling large free-text input, however research on their performance in this setting is still limited [20]. In parallel, integration of data from wearable devices such as heart rate, temperature and oxygen saturation, could support further advances and improvements in the accuracy of symptom checkers.

Secondly, OSCs and PCPs may fundamentally differ in their approach. PCPs may use the “rule out worst-case scenario” approach, especially in the context of minor illness, utilising red flags to exclude more serious illness [13, 21]. Whereas, OSCs may seek the most probable cause of a patient symptoms, and given the high incidence of minor illness, overlook seeking rare but important causes. This important discrepancy may reflect differences in approach, not reflected in measures of accuracy. For example, non-specific lower back pain is the most common cause of back pain symptoms, accounting for 95% of lower back pain presenting to primary care [22], OSCs may appear highly accurate at diagnosing back pain, simply due to the underlying frequency of the condition. However, by not seeking red flags, they differ in approach to PCPs and do not reflect current clinical guidance [9]. 

Reassuringly, despite the lack of red flags sought, when a condition was accurately diagnosed by the OSC, additional reading to the user was presented which typically included descriptions of red flag symptoms that users should be aware of.

### Study limitations

We encountered several limitations. Firstly, despite their widespread use, red flags lack universal definitions and may vary across similar clinical presentations [23]. Secondly, our small selection of OSCs limit the generalisability of our findings and increase the possibility that our findings are due to chance. Thirdly, individual differences in OSCs performance indicate that OSCs cannot be treated as a single group. Differences in performance may reflect differences in design and development and given OSCs are evolving, current research findings may not reflect current performance. The generalisability of our findings is also limited due to the use of vignettes, rather than real world data. Typically, vignettes are written by clinicians and may not reflect real world clinical complexity, or the language and description used by patients. Further research using real-world data would aid in future evaluation studies. Finally, critically we were unable to test whether in the presence of red flags symptoms, OSCs altered the advice given. This would ensure that OSCs not only seek relevant red flag symptoms but also respond appropriately when they are present.

### Study implications

To date, no previous studies have examined OSCs coverage of red flag symptoms. Prior evaluations of OSCs have focused on diagnostic and triage accuracy. However, such evaluations may overlook critical aspects of primary care consultations, where exclusion of serious conditions through seeking of red flag symptoms may take precedence over accuracy of diagnosis. This process of seeking red flags is an important component of primary care consultations and is used as a marker of competency in the assessment of doctors training in primary care, such as those undergoing assessment by the Royal College of General Practice in the UK [24]. 

Despite concerns around the safety of symptoms checkers [25, 26], their use is likely to grow, especially given the current challenges facing primary care both in the UK and globally [2, 27, 28], with difficulty obtaining appointments and falling patient satisfaction [29, 30]. Digital technology has been proposed to help alleviate some of the challenges faced [31, 32], with the potential to reduce demand [33]. However further research is needed to ensure their safety and utility in primary care settings.

## Conclusion

Previous OSCs evaluations overlook critical areas of safety relevant to primary care, such as red flag identification. This is particularly relevant in primary care, where ruling out serious illness is a key component of consultation. Our findings suggest that OSCs in their current form do not adequately seek red flags. Future research should address these gaps to ensure OSCs can be safely integrated into primary care settings.

## Supplementary Information

Supplementary Material 1

## Data Availability

The datasets used and/or analysed during the current study are available from the corresponding author on reasonable request.

## Abbreviations

OSCs: Online symptom checkers
PCPs: Primary care physician
