# Supplementary material for: Evaluating the use of red flags by online symptom checkers
Source: BMC Health Serv Res. 2025 Oct 1;25:1263. doi: 10.1186/s12913-025-13353-w (PMC12486864; doi:10.1186/s12913-025-13353-w)
Supplement: Supplementary file 1 — Supplementary Material 1 [file 12913_2025_13353_MOESM1_ESM.docx]

# Appendix

Table 1. Clinical Vignette Classification by standard setters.

|  | PCPs 1 | PCPs 2 | Consensus |
| --- | --- | --- | --- |
| Vignettes for emergency triage | 14 | 15 | 14 |
| Vignettes for Primary Care triage | 37 | 36 | 37 |

Table 2. Red flags among 37 primary care triage vignettes determined through consensus by standard setters.

| Percentage of primary care triage vignettes with 1 or more red flags | 67.6 % (25) |
| --- | --- |
| Mean (Range) of red flags among primary care vignettes | 2.6 (0-8) |
| Total number of red flags | 77 |

Table 1. Example of clinical vignettes by triage

| **Triage** | **Vignette** |
| --- | --- |
| Non-emergency triage | 35 year male, with 3 month history of lower back pain, worse on bending |
|  | 30 year old male with 2 days of fever, body ache and headache |
|  | 18 year old female with 1 week history of fever, sore throat, fatigue, pain on swelling and fever |
|  | You are Jason King, a lorry driver, and have come to see the doctor about your nails. All finger and toenails are affected. They have been like this for many years. They are not painful and crumble easily. Information divulged if asked specifically: Your skin and scalp psoriasis is well controlled. |
|  | A 52 year old man and has been using a dating app for the first time since his divorce.  He has met several new partners and has been spending the night with some of them.  He has been complaining of stinging when passing urine for the last week. |
| Emergency Care Triage | 64 y/o m, 1 day chest pain (8/10), non-radiating substernal chest pressure, sweating, shortness of breath, (chest tightness) |
|  | 54 y/o M sudden,  painless, complete loss of vision left eye 10 hours ago, history of diabetes, hypertension, and high cholesterol |
|  | A 21 year old man has been getting more short of breath over the past 1 hour.  10 puffs of his salbutamol inhaler hasn't helped.  He does not have chest pain.  He has had a dry cough for the past few days but now is very wheezy.  He is struggling to breathe and cannot speak a full sentence. |
